# Understanding a successful obesity prevention initiative in children under 5 from a systems perspective

**DOI:** 10.1371/journal.pone.0195141

**Published:** 2018-03-29

**Authors:** Brynle Owen, Andrew D. Brown, Jill Kuhlberg, Lynne Millar, Melanie Nichols, Christina Economos, Steven Allender

**Affiliations:** 1 Deakin University, Global Obesity Centre (GLOBE), Centre for Population Health Research, School of Health and Social Development, Faculty of Health, Geelong, Victoria Australia; 2 North Carolina State University, Department of Agriculture and Human Sciences, NC Cooperative Extension, Raleigh, NC United States of America; 3 Australian Health Policy Collaboration, Victoria University, Melbourne, Victoria Australia; 4 Friedman School of Nutrition Science and Policy, Tufts University, Boston, MA United States of America; Vanderbilt University, UNITED STATES

## Abstract

**Introduction/Background:**

Systems thinking represents an innovative and logical approach to understanding complexity in community-based obesity prevention interventions. We report on an approach to apply systems thinking to understand the complexity of a successful obesity prevention intervention in early childhood (children aged up to 5 years) conducted in a regional city in Victoria, Australia.

**Methods:**

A causal loop diagram (CLD) was developed to represent system elements related to a successful childhood obesity prevention intervention in early childhood. Key stakeholder interviews (n = 16) were examined retrospectively to generate purposive text data, create microstructures, and form a CLD.

**Results:**

A CLD representing key stakeholder perceptions of a successful intervention comprised six key feedback loops explaining changes in project implementation over time. The loops described the dynamics of collaboration, network formation, community awareness, human resources, project clarity, and innovation.

**Conclusion:**

The CLD developed provides a replicable means to capture, evaluate and disseminate a description of the dynamic elements of a successful obesity prevention intervention in early childhood.

## Introduction

The prevalence of overweight and obesity among children continues to increase [1–3] with concomitant negative implications for long-term morbidity and mortality in adulthood.[4, 5] The high adult burden [6] and developmental profile of adult obesity reinforce the need for prevention during childhood and particularly in early childhood.[7]

Setting-based approaches have shown some promise in preventing the onset of obesity [8, 9] though this effect appears to recede once programmatic funding is removed. [10, 11] The main critique of these interventions is that they are focused on a single setting, usually pre-schools or health care, and overlook the broader complexity of environmental and social determinants of obesity. [10, 11] Successful interventions in the United States and Australia [8, 9, 12–15] have taken a broader community view and oriented prevention efforts across multiple community sectors and settings. These trials support current calls for childhood obesity prevention interventions to actively engage at all levels of a community and apply multiple strategies based on a shared understanding of the numerous drivers inherent in each community context.[16]

Systems science has emerged as a discipline to identify, understand and organize the drivers of complex problems [17, 18] including obesity [19], and has the potential to support and underpin interventions.[20] System dynamics (SD) is one discipline within systems science that provides tools to capture and understand the complex behaviours of a system.[21, 22] One specific tool from SD, called a causal loop diagram (CLD),[23, 24] provides a shared understanding of the many drivers of complex problems and relationships between them.

A key tenet of systems thinking is to understand the underlying non-linear structure of systems and the ways in which feedback and delays dictate overall system behaviours.[25, 26] When feedback loops are in operation within a system, and a change is made in a system, underlying mechanisms in the system feedback and impact the original point of change. Reinforcing feedback loops, often associated with virtuous and vicious cycles, amplify change and produce behaviour patterns of exponential growth and decay. Virtuous cycles involve the amplification of positive change in a system, such as funding, staff skills, or community engagement, and the decay of negative factors such as resistance to change, staff turnover, or negative health outcomes. Vicious cycles are the opposite, where negative changes are amplified and positive changes decay. Any reinforcing feedback cycle represented in a causal loop diagram could operate as a virtuous or a vicious cycle depending on the point in time and the conditions in the rest of the system. Balancing feedback loops work to counteract change in a system, limiting its growth and slowing its decline. For example, if an organisation receives grant funding to implement a new health program, there will be mechanisms that reinforce the organisation’s ability to implement programs, such as leveraging successes from the health program to apply for further grant funding, and there will be balancing mechanisms that limit an organisation’s success such as resistance from staff to changes that result from implementing new programs. These reinforcing and balancing effects make implementation and sustainability of a new health program considerably more complex.

The 2007 Foresight Obesity Systems Atlas was one of the first causal loop diagrams to demonstrate the complex causes of obesity and their interactions.[27] The Foresight project brought together many of the world’s leading obesity experts in an attempt to generate a comprehensive representation of all of the factors relevant to obesity for individuals and populations, their relationships and interdependencies. The resulting ‘obesity systems map’ presents a causal model that begins with energy balance at an individual level and builds a peripheral set of 108 variables that directly or indirectly influences energy balance. Given the complexity of obesity, obesity prevention project implementation itself is complex, with many interacting factors and feedback loops supporting or hindering implementation success. Large scale intervention studies commonly collect detailed formative, process and outcome data as part of standard evaluations, across multiple levels of the system. In many cases these data sets could be further exploited to generate system insights, to explicate the role of systems and system elements in driving the success or otherwise of these interventions, provide a deeper understanding of how the intervention functioned within the system, and to support prospective intervention design which takes an explicitly system oriented approach. This study leverages an available dataset to pilot using systems perspectives and analyses. The aim of this study was:

to develop a causal loop diagram to represent and better understand the dynamic changes of project implementation over time of a successful community-based obesity prevention intervention in children under 5 and generate from this example a general process that can be applied to other projects in public health.

## Methods

### Data and sample

Data from the Romp & Chomp Project [28, 29] were retrospectively re-analysed to create the CLD. Romp & Chomp was a trial of a multi-setting, multi-strategy community-based obesity prevention intervention targeting 12,000 children aged 0–5 years, conducted in a large regional city (Geelong, Victoria Australia) from 2004 to 2008. The intervention focused on community capacity building and environmental (policy, sociocultural, economic and physical) changes to increase healthy eating and active play in multiple early childhood care and educational settings. The Romp & Chomp evaluation showed a significant impact on overweight and obesity; following the intervention, compared to the control group, there was a significantly lower mean weight, BMI, and BMI z-score in the 3.5-y-old children and a significantly lower prevalence of overweight and obesity in both the 2- and 3.5-y-old children.[9]

### Data analysis

Semi-structured interviews seeking information on project implementation and sustainability were conducted at the completion of Romp & Chomp in 2008 with 16 stakeholders including community health workers, long day care staff and the project’s steering committee and management committee members.[29] Based on initial analysis of the interviews, project implementation was identified as the dynamic variable of interest for the construction of a causal loop diagram (CLD). The dynamic behaviour of project implementation was described as increasing over time, meaning the various activities of the project were developed and implemented over time until the project successfully achieved its aim (as measured by the impact on child weight status). The goal of building a CLD was to further describe and understand the feedback loops that led to the success of project implementation. The initial CLD was constructed from the transcripts of these key informant interviews. The data were reviewed until data saturation was reached, whereby subsequent review of interviews added nothing new.

We followed a systematic method to derive causal structures from interview transcripts described by Kim and Andersen [30] and which draws on grounded theory and associated coding strategies.[31] This approach has been used both in studies where data were gathered explicitly for building CLD and where data were initially collected for other purposes.[30, 32] Transcripts were first open-coded by identifying text that explicitly described or implied causal linkages between two concepts (hereon called variables) (see step 1, Table 1 for example). Each fragment of text was translated to microstructures describing cause variables, effect variables, and relationship polarity (e.g. step 2, Table 1). Two researchers collaborated on the process of identifying the structures to reduce bias in interpreting causal relationships in the data.

**Table 1:** Example coding chart to inform CLDs from interview transcripts.

Step 1 Coded text showing causal linkage			
Text: “The turnover of the staff made coordination difficult from time to time, so when they hired a full-time R&C staff, coordination improved.” Informant ID: 1			
Step 2 Translation to microstructure(s)			
	Cause variable	Effect variable	Relationship type
Microstructure 1	Staff turnover	Coordination of partners	Negative
Microstructure 2	Hiring full-time staff	Coordination of partners	Positive
Step 3 Causal statements drawn into words-and-arrow diagrams			
Cause	Effect	Relationship type	Words-and-arrow diagrams
Staff turnover	Coordination of partners	-	Staff turnover →- Coordination of partners
Hiring full time R&C staff	Coordination of partners	+	Full time R&C staff →+ Coordination of partners

Adapted from Kim & Anderson [30]

System dynamics conventions were used to identify causal relationships. A positive polarity (represented as ‘+’) indicates a positive relationship between the two variables (i.e., as cause increases, the effect increases and as cause decreases, the effect decreases), a negative polarity (-) indicates an inverse relationship between the two variables (i.e., as cause increases, effect decreases and as cause decreases, effect increases). A dash sign (//) indicates an element of delay in effect, relative to the time scale of the remainder of the diagram. This nomenclature was used to develop a graphical representation of individual cause and effect relationships within the CLD (step 3). Each of these graphical representations were collated into a composite map representing all microstructures using Vensim (Ventana Systems, Harvard, MA). The initial map was further refined by connecting microstructures with the same variables (i.e. repeated in several microstructures), removing unnecessary duplicate variables and combining equivalent variables under a single variable name. The emerging causal structures were repeatedly verified by returning to the context of the verbatim text alongside causal structures and variable behaviour.

The resulting initial CLD was large and unwieldy and a further filter was applied to remove exogenous variables (that is, variables not within a feedback loop) and remove those that were not within the influence of the Romp & Chomp group. Exogenous variables were removed because of the choice to focus on feedback loops, one of the key ways to understand system change over time in system dynamics.[33] A constant comparative approach was used in comparing the result of each change against the original diagram to ensure fidelity of meaning and further attention was paid to ensure a focus on identifying feedback loops. Specific feedback loops were identified as key areas of the data corpus that were highlighted as important by the interviews. Two experts who were actively involved in the implementation of Romp & Chomp reviewed the emerging causal loop diagram to validate the ongoing changes and final diagram. They considered the names of the variables and the overall feedback loops. They considered whether the narrowed down variables and feedback loops matched their experience with Romp & Chomp and the results of other analyses of available data from the project.

## Results

**Fig 1:**
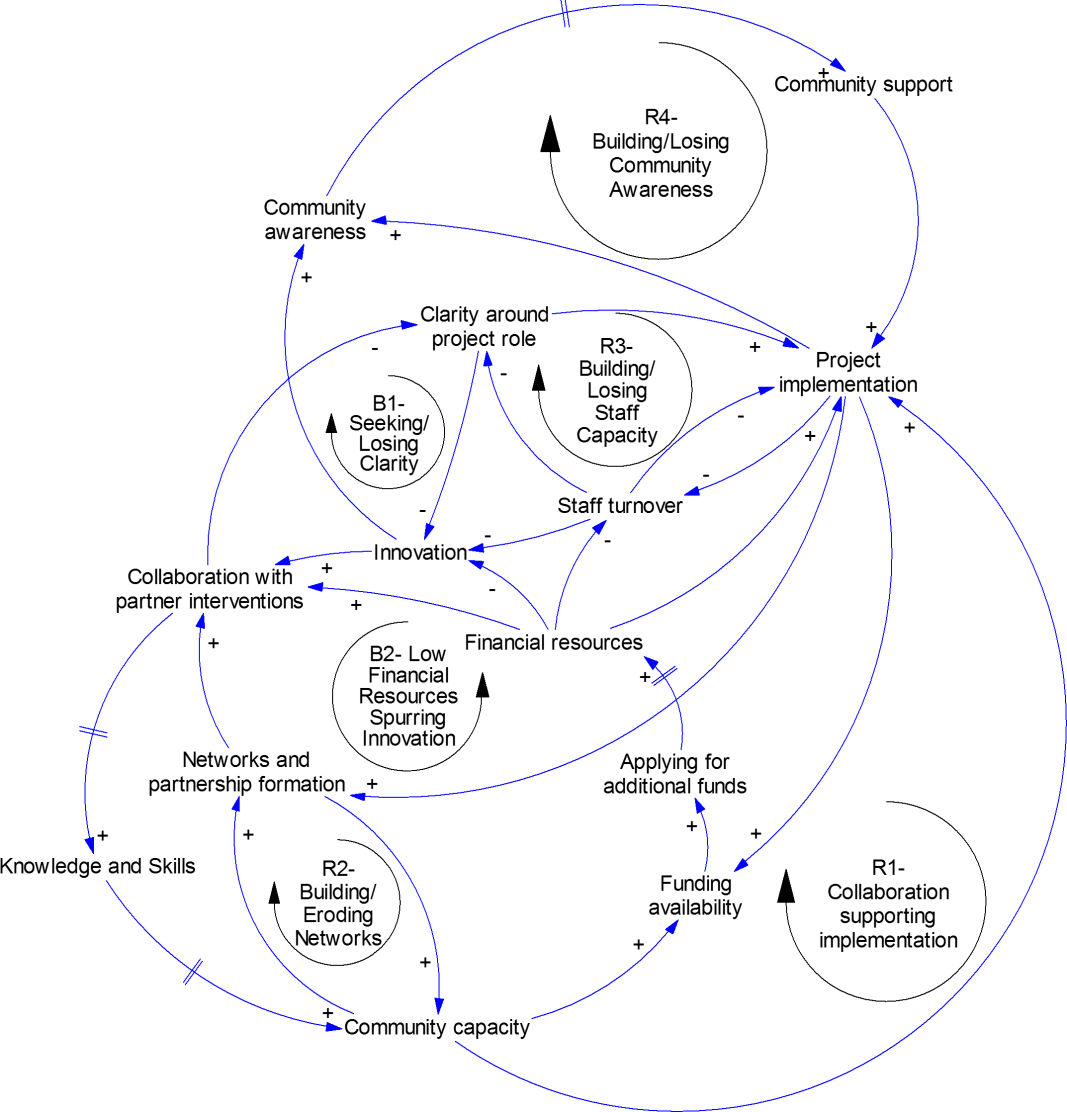
Causal loop diagram of Romp & Chomp.

The final CLD (Fig 1) contained six key feedback loops explaining changes in the project implementation of Romp & Chomp over time. Project implementation followed a pattern where it steadily increased at first, followed by a slow down as barriers such as staff turnover and role clarity interfered, but ultimately continued successfully due to the role played by innovation. Each of the loops are described below.

### Detail of feedback loops

#### R1 (reinforcing loop 1)–Collaboration supporting implementation

As initial *project implementation* began, *networks and partnership formation* grew as stakeholders worked together on Romp & Chomp. As networks formed, *collaboration with partner interventions* strengthened. As partners collaborated and engagement in the project increased, this created opportunities for community members and health workers to gain new *knowledge and skills* in health promotion. After a delay, *community capacity* increased as *knowledge and skills* spread across organisations. This increasing of *community capacity* then enhanced *project implementation*, creating a virtuous cycle.

#### R2 –Building/Eroding networks

As *community capacity* increased, this led to *networks and partnership formation*. The strength of these *networks and partnership formation* then increased *community capacity* over time, leading to a virtuous cycle.

#### B1 (balancing loop 1)—Seeking/Losing clarity

One trade off to the growth in networks working together on Romp & Chomp was a loss of clarity in project role. As *collaboration* increased, with both an increasing number of organisations involved and engagement from an increasing number of individuals within participating organisations, *clarity around project role* decreased, as people struggled to keep track of their part of the project. This decrease in clarity then hindered *project implementation*, creating an increase in *staff turnover* as people’s frustration grew. Greater *staff turnover* limited opportunities for *innovation*, which hindered *collaboration*, putting a limit on how much *collaboration* was possible.

#### R3- Building/Losing staff capacity

The effort put into Romp & Chomp by the coordinators was core to successful project implementation. As *financial resources* began to decrease however, this started to create *staff turnover*. With greater *staff turnover* came even less *clarity around project role*, creating further challenges to *project implementation*, compounding the dynamic of increasing *staff turnover*.

#### B2- Low financial resources spurring innovation

Innovation was the key solution to the problems created by *financial resources* depleting. As the initial *financial resources* decreased and began to run out, this spurred an increase in *innovation* as the implementation team found ways to keep Romp & Chomp going with limited resources. A key outcome of *innovation* was further *collaboration with partner innovations*, supporting further *community capacity building*. With increased *community capacity building*, *funding availability* increased, as opportunities to fund specific innovative aspects of the project arose. The Romp & Chomp team *applied for additional funds*, but there was a very long delay before that actually led to increased *financial resources*. As a result, while some *financial resources* did come in slowly, it was ultimately insufficient to maintain external investment in the project. This balancing loop also suggests that an excess of funding can limit innovation and collaboration, and over time, reduce new funding awarded.

#### R3- Building/Losing community awareness

The increase in *innovation* spurred by limited financial resources had an additional positive effect. As the staff innovated, this increased *community awareness* of Romp & Chomp, as members of the community took notice of the interesting activities happening as part of the intervention. Over time, this increased *community awareness* increased *community support*, leading to better *project implementation* and further *innovation*, continuing the cycle of growing community awareness.

## Discussion

New methods that incorporate systems thinking provide opportunities to strengthen community based health promotion programs throughout the project lifecycle, from planning through implementation and evaluation. This study presents a strong, systematic method to visually represent the dynamic drivers of a community based obesity prevention intervention. This analysis shows that historical intervention process data can offer insights into the drivers and barriers to intervention success or failure in a way that reflects the complexity of a successful obesity intervention in early childhood.

To date childhood obesity prevention efforts have primarily been driven by linear logic models.[34–36] The 2015 Lancet Obesity Series,[37] described the central challenge in combatting childhood obesity as creating sustained, large-scale, community-based interventions that tackle complexity and work at multiple levels. Systems science appears the most promising approach for addressing this complexity, because it facilitates consideration of interactions among such broad-ranging obesogenic factors as individual behaviours, government and organisational policies, as well as social, built, natural, and economic environments. Systems science has been successfully applied in other fields but as yet there are few examples in obesity prevention of approaches that analyse the complex drivers of obesity and related implementation challenges.[24, 37] Testing and learning from approaches such as the method described here represent new, analytically informed ways to strengthen existing systems or create new ones [38] and point to novel ways to promote healthy weight and prevent obesity and associated conditions.

This method has provided a number of insights into the ways in which a successful community-based obesity prevention intervention in early childhood functioned from a systems perspective. The CLD demonstrates how factors impacting project implementation can be considered from a feedback perspective, as opposed to traditional models. Thinking in terms of feedback can lead to more effective, sustainable intervention design in the future by deepening understanding of unintended consequences, building logic models that go beyond simple linear cause and effect from inputs to outcomes, and suggesting places where change may be exponential, characterised by slow change initially that accelerates.[33]

### Strengths and limitations

A strength of the method was the repeated triangulation of emerging causal structures by returning to the context of the verbatim text alongside causal structures and variable behaviour. The approach openly and intentionally involved a collaborative effort of two researchers to reduce bias in interpreting causal relationships in the data. Expert opinion from those central to the Romp & Chomp intervention was sought and used to validate the emerging causal loop diagram. A second strength of the method was the use of multiple interviews with key intervention personnel; integrating these perspectives increased the range of data to inform an understanding of the underlying system drivers involved with the Romp & Chomp intervention.

While the final CLD provides one graphical representation of the key feedback loops in the system that drove the Romp & Chomp intervention, it must be interpreted in the context of the data that informed its construction. The CLD was developed from retrospective examination of secondary data (key stakeholder interviews), collected for other evaluation purposes.[29] The original interview questions sought to examine community capacity across four domains, including network partnerships, knowledge transfer, problem-solving and infrastructure. Thus, the model resulting from analysis of these interviews may be biased to reflect these elements and may also fail to capture system elements not addressed in the interviews. While these are legitimate limitations the intention here is to offer a process by which investigators can engage with, and apply, systems thinking at the evaluation phase of practice.

The systematic method used to capture causal relationships from qualitative text data in a CLD is consistent with existing approaches.[32] Our method was based on the one proposed by Kim and Andersen [30] and the methods provide a technique to retrospectively evaluate community interventions from a systems perspective and understand the way successful and unsuccessful interventions addressed complexity. These lessons can then be applied prospectively to increase the chances of success for new prevention initiatives. For practitioners, it has the potential to provide insight into community-wide systems and potential leverage points to target and structure community interventions.

### Further research and unanswered questions

Further work is required to examine whether this functions as an effective evaluation method to understand the complexity of whole of system interventions. Further questions raised by this study include how to assess the validity of a CLD that has been developed to describe how an intervention has functioned or will function within a community. While the approach is qualitative in nature, the large datasets associated with major interventions such as EPODE,[39] OPAL,[40] and Healthy Together Victoria,[41] represent an exceptional opportunity to elicit lessons about community interventions, although currently, the resources required to conduct such analysis would be onerous. Combining the techniques applied here with machine learning techniques [42] may provide the means to undertake such analysis at scale, enhance generalisability and expand the evidence base to bring the masses of process data, which are usually underutilised, further to bear on future intervention efforts.

## Conclusion

This paper demonstrates it is possible to create a representation of the complexity of community based interventions from retrospective analysis of process data. Creating this representation allows interventions to be understood from the perspective of feedback loops and delays, as opposed to traditional linear logic models. These techniques coupled with traditional approaches to intervention design, implementation and evaluation provide an extension to the toolbox for community based obesity prevention.
